# Blockade of Nuclear *β*‐Catenin Signaling via Direct Targeting of RanBP3 with NU2058 Induces Cell Senescence to Suppress Colorectal Tumorigenesis

**DOI:** 10.1002/advs.202202528

**Published:** 2022-10-21

**Authors:** Can‐Can Zheng, Long Liao, Ya‐Ping Liu, Yan‐Ming Yang, Yan He, Guo‐Geng Zhang, Shu‐Jun Li, Ting Liu, Wen Wen Xu, Bin Li

**Affiliations:** ^1^ Key Laboratory of Biological Targeting Diagnosis Therapy and Rehabilitation of Guangdong Higher Education Institutes The Fifth Affiliated Hospital of Guangzhou Medical University Guangzhou Medical University Guangzhou 510799 China; ^2^ MOE Key Laboratory of Tumor Molecular Biology and Key Laboratory of Functional Protein Research of Guangdong Higher Education Institutes Institute of Life and Health Engineering Jinan University Guangzhou 510632 China; ^3^ Key Laboratory of Protein Modification and Degradation School of Basic Medical Sciences Guangzhou Medical University Guangzhou 511495 China

**Keywords:** *β*‐catenin, cell senescence, colorectal cancer, Ran‐binding protein 3, small molecule compounds

## Abstract

Colorectal cancer (CRC) is one of the most common malignant tumors in the world, with high prevalence and low 5‐year survival. Most of the CRC patients show excessive activation of the Wnt/*β*‐catenin pathway which is a vital target for CRC treatment. Based on multiple CRC cell lines with different nuclear expression of *β*‐catenin, NU2058 is identified from a small molecule library consisting of 280 bioactive compounds and found to selectively inhibit the proliferation of CRC cells with nuclear *β*‐catenin activation in vitro and in vivo. The translational significance of NU2058 alone or in combination with chemotherapeutic drugs oxaliplatin and irinotecan (SN38) in CRC is demonstrated in orthotopic tumor model and patient‐derived xenograft models. By integrating limited proteolysis‐small molecule mapping (LiP‐SMap) and mass spectrometry (MS), Ran‐binding protein 3 (RanBP3) is identified as the direct target of NU2058. The results show that RanBP3 is a tumor suppressor in CRC and is associated with patient survival. Mechanistically, NU2058 increases the interaction of RanBP3 and *β*‐catenin to promote nuclear export of *β*‐catenin, which further inhibits transcription of c‐Myc and cyclin D1 to induce cell senescence. Collectively, NU2058 may serve as a promising therapeutic agent for CRC patients with selective disruption of pathologic Wnt/*β*‐catenin signaling.

## Graphical abstract

Colorectal cancer (CRC) is one of the most common malignant tumors. Most of the CRC patients show excessive activation of Wnt/*β*‐catenin pathway which is a vital target for CRC treatment. Ran‐binding protein 3 (RanBP3) may be a promising prognostic biomarker and therapeutic target in CRC, and a preclinical rationale is provided for NU2058 as a potential strategy for cancer treatment.

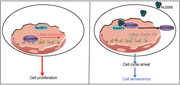

## 1 Introduction

Colorectal cancer (CRC) is the third leading cause of cancer‐related death in the world, with an estimated 1.9 million new cases and 935 000 deaths in 2020, accounting for approximately one‐tenth of cancer cases and deaths.^[^ ^1^ ^]^ In addition to the high incidence, the limited choice of chemotherapeutic drugs also contributes to the high mortality rate of CRC patients.^[^ ^2^ ^]^ There is an urgent need to develop effective and low‐toxicity drugs to combat CRC. More than 90% of CRC patients have constitutive activation of the Wnt/*β*‐catenin pathway due to adenomatous polyposis coli (APC) or *β*‐catenin mutations, which disrupts the cytoplasmic *β*‐catenin destruction complex and leads to the nuclear translocation of *β*‐catenin. Nuclear *β*‐catenin binds to the transcription factors T‐cell factor (TCF)/lymphoid enhancer factor binding factor and some coactivators to form a transcription‐promoting complex, increasing the transcription of Wnt‐related targets, including c‐Myc and cyclin D1, which promote CRC tumorigenesis.^[^ ^3^ ^]^ The activation of nuclear *β*‐catenin signaling is crucial for the development and progression of CRC, and nuclear *β*‐catenin signaling is an important target for CRC therapy.^[^ ^4^ ^]^ However, the development of inhibitors targeting the Wnt/*β*‐catenin signaling pathway with satisfactory effectiveness and low toxicity is still underway.^[^ ^5^ ^]^

In this study, a bioactive compound library consisting of 280 small molecules was screened to identify compounds with anticancer bioactivities against two CRC cell lines: DLD1, which has high nuclear *β*‐catenin expression, and RKO, which has low nuclear *β*‐catenin expression. A normal colonic epithelial cell line, NCM460, was also used for screening. NU2058 was found to significantly suppress the growth of DLD1 cells but not RKO cells without significant toxicity against NCM460 cells, and therefore attracted our interest. NU2058 was first identified as a guanine‐based cyclin‐dependent kinase (CDK) (CDK1/CDK2) inhibitor.^[^ ^6^ ^]^ Interestingly, NU2058 was also reported to exert a CDK2‐independent effect on cancer chemoresistance.^[^ ^7^ ^]^ To date, the anticancer bioactivity of NU2058 in CRC and the underlying mechanism have not been reported.

By integrating RNA sequencing (RNA‐seq) and Kyoto Encyclopedia of Genes and Genomes (KEGG) analysis, the pathways enriched by differentially expressed genes in NU2058‐treated DLD1 and RKO cells were compared. Senescence was proposed to account for the molecular mechanisms underlying the anticancer effect of NU2058 in CRC cells with nuclear *β*‐catenin activation. Cellular senescence is a cellular defense mechanism that protects cells from many types of damage, including oncogene activation and oxidative and genotoxic stress.^[^ ^8^ ^]^ Although cellular senescence has been identified as a permanent state of cell cycle arrest in proliferating cells and an initial barrier for cancer development,^[^ ^9^ ^]^ the signaling mechanisms that trigger cellular senescence remain unclear.

In this study, we aimed to examine the anticancer properties of NU2058 in multiple cell lines and orthotopic and patient‐derived xenograft (PDX) models and to identify the direct target(s) of NU2058 by limited proteolysis‐small molecule mapping (LiP‐SMap) and mass spectrometry (MS). The biological and clinical significance of Ran‐binding protein 3 (RanBP3) was studied in CRC, and whether NU2058 directly binds to RanBP3 and disrupts RanBP3‐*β*‐catenin‐c‐Myc/cyclin D1 signaling was investigated. The results of this study will open new rational avenues for therapeutic interventions for CRC patients.

## 2 Results

### 2.1 NU2058 Selectively Suppresses the Tumorigenesis of CRC Cells with Nuclear *β*‐Catenin Activation In Vitro and In Vivo

**Figure 1:**
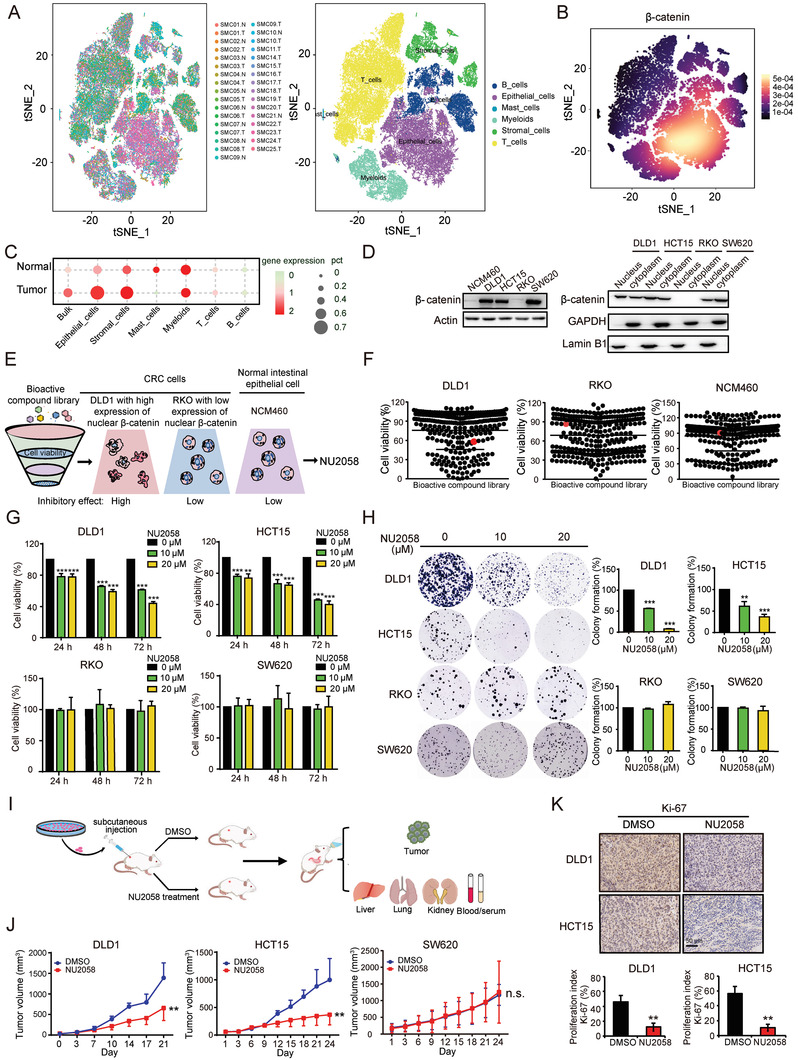
NU2058 selectively suppresses the tumorigenesis of CRC cells with nuclear *β*‐catenin activation in vitro and in vivo. A,B) The t‐SNE analysis showed concentration of *β*‐catenin expression in epithelial cells. C) *β*‐catenin was upregulated in bulk cells, epithelial cells, and stromal cells of tumors compared with normal tissue. D) Western blotting showing the expression of *β*‐catenin in the nucleus and cytoplasm of NCM460, DLD1, HCT15, RKO, and SW620 cells. E) Experimental diagram illustrating the strategy used to identify candidate drugs from a library consisting of 280 bioactive compounds. F) DLD1, RKO, and NCM460 cells were exposed to the 280 bioactive compounds (10 µm, 72 h) individually and subjected to WST‐1 assay. G) DLD1, HCT15, RKO, and SW620 cells were treated with NU2058 at increasing concentrations (0, 10, and 20 µm) for 24, 48, and 72 h, and WST‐1 assay was performed to determine cell viability. H) Colony formation assay showing the abilities of CRC cells to form colonies when exposed to the indicated concentrations of NU2058. I) Experimental strategy for the establishment of tumor xenografts and drug treatment. J) The growth of tumor xenografts established from DLD1, HCT15, or SW620 cells was monitored. Images of tumors excised from six mice 24 days after inoculation (*n* = 6). K) Ki‐67 immunohistochemical staining indicated that cell proliferation was inhibited upon NU2058 treatment (*n* = 3). Bars, SD; *p < 0.05; **p < 0.01; ***p < 0.001; compared with cells treated with DMSO.

To investigate the heterogeneous expression of *β*‐catenin in CRC tumors, we analyzed a single‐cell RNA‐sequencing (scRNA‐seq) dataset from UCSC Cell Brower (https://cells.ucsc.edu/).^[^ ^10^ ^]^ Compared with traditional transcriptome sequencing, scRNA‐seq is a powerful technique for dissecting the heterogeneity of solid tumors.^[^ ^11^ ^]^ The t‐distributed stochastic neighbor embedding (t‐SNE) analysis and the density plot of *β*‐catenin revealed that *β*‐catenin was mainly expressed in epithelial cells compared with stromal cells (**Figure** **1**A,B). As expected, *β*‐catenin was indeed significantly upregulated in epithelial cells of tumors compared with normal tissues (Figure 1C). The above findings inspired us to search for new anticancer drugs with the potential to suppress CRC tumorigenesis, in particular, those that could selectively inhibit the activation of nuclear *β*‐catenin. Based on a small molecule drug library consisting of 280 bioactive compounds, two CRC cell lines (DLD1, which has high expression of nuclear *β*‐catenin, and RKO, which has low expression of nuclear *β*‐catenin) as well as one normal intestinal epithelial cell line (Figure 1D) were subjected to WST‐1 assay (10 µm, 72 h) for cell viability analysis (Figure 1E,F and Table S1, Supporting Information). NU2058, an inhibitor of CDKs or DNA topoisomerase II, was found to exert a significant inhibitory effect on DLD1 cells but a moderate effect on NCM460 and RKO cells, and thus became our focus for further experiments (Figure 1F). To further evaluate the effect of NU2058 on CRC cell proliferation, DLD1, HCT15, RKO, and SW620 cells were treated with increasing concentrations of NU2058 for different durations. As shown in Figure 1G, NU2058 significantly inhibited the proliferation of DLD1 and HCT15 cells, CRC cell lines with high expression of nuclear *β*‐catenin, in a dose‐ and time‐dependent manner, but did not have this effect on RKO and SW620 cells, which have low expression of nuclear *β*‐catenin. Consistently, the abilities of DLD1 and HCT15 cells to form colonies were markedly reduced by NU2058 treatment, but the number of RKO‐ and SW620‐forming colonies was not affected (Figure 1H).

To further evaluate the anticancer effect of NU2058 in vivo, subcutaneous tumor xenografts were established in nude mice, and the animals were orally administered NU2058 (15 mg/kg/day) or vehicle (Figure 1I). As shown in Figure 1J, the volumes of the xenografted tumors generated by DLD1 and HCT15 cells were reduced by 53% and 62% after exposure to NU2058 treatment. However, there was no change in SW620 cell‐derived tumor xenografts. Ki‐67 immunohistochemical staining indicated that NU2058 significantly suppressed the proliferation index of tumor xenografts generated from DLD1 and HCT15 cells (Figure 1K). We also noted that there was no significant difference in terms of body weight, critical organ histological features, serum levels of aspartate aminotransferase (AST) and alanine aminotransferase (ALT), or various blood indexes between the treatment and control groups (Figure S1A–C, Supporting Information). Furthermore, we determined whether NU2058 could exhibit anticancer bioactivity in other cancers, and the results of WST‐1 assay showed that NU2058 also inhibited cell proliferation in esophageal and liver cancer cells (Figure S1D, Supporting Information). In summary, we propose that NU2058 is a potential novel anticancer agent with low toxicity for CRC patients with *β*‐catenin signaling activation.

### 2.2 NU2058 Disturbs the Nuclear Translocation of *β*‐Catenin and Transcription of *cyclin D1* and *c‐Myc*

**Figure 2:**
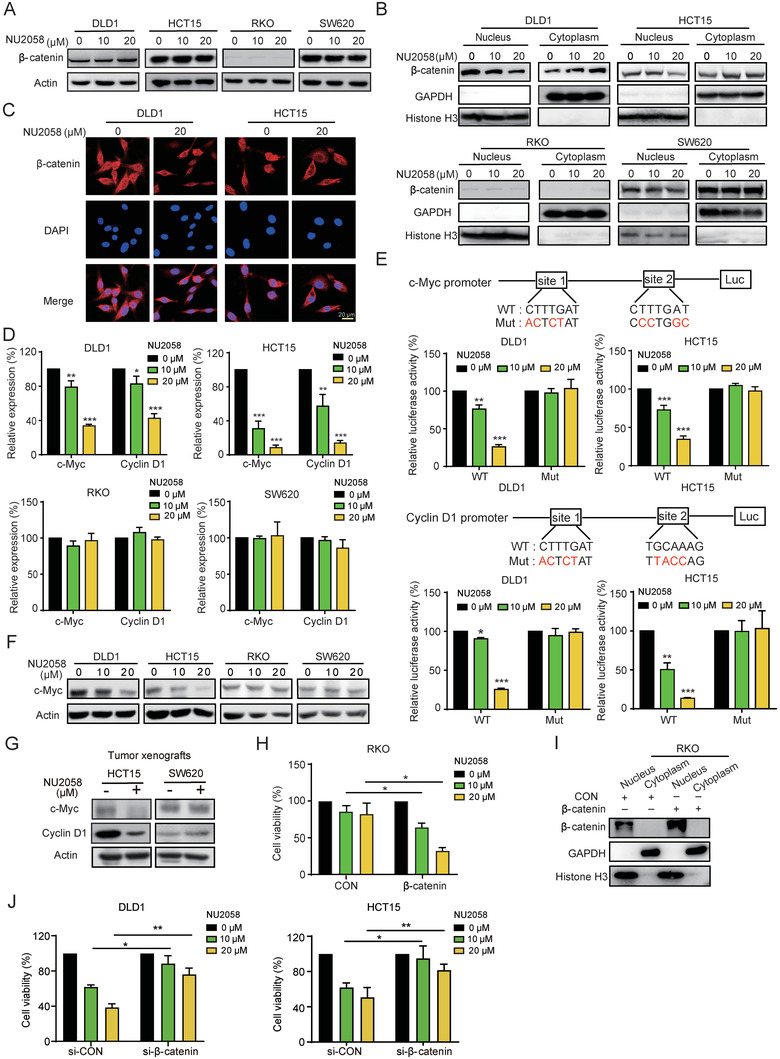
NU2058 disturbs the nuclear translocation of *β*‐catenin. A) The total *β*‐catenin level was detected in NU2058‐treated (0, 10, and 20 µm, 72 h) CRC cells. B) Subcellular fractionation and Western blotting were used to assess the expression of *β*‐catenin in the nucleus and cytoplasm of CRC cells exposed to the indicated concentrations of NU2058. C) Immunofluorescent staining showing the subcellular localization of *β*‐catenin in CRC cells treated with NU2058 (0 and 20 µm, 72 h). D) qRT–PCR was used to detect the mRNA levels of cyclin D1 and c‐Myc in NU2058‐treated CRC cells. E) Design of the mutated cyclin D1 and c‐Myc promoters. Dual luciferase reporter assay was performed to detect the effect of NU2058 on the activity of the cyclin D1 or c‐Myc promoter in CRC cells. F) Western blotting showing that cyclin D1 and c‐Myc expression was inhibited by NU2058 (0, 10, and 20 µm) in DLD1 and HCT15 cells. G) Western blotting analysis of cyclin D1 and c‐Myc expression in NU2058‐treated tumor xenografts. H) WST‐1 assay illustrating that forced expression of *β*‐catenin in RKO cells increased their sensitivity to NU2058 (0, 10, and 20 µm, 72 h). I) Detection of the expression of *β*‐catenin in the nucleus and cytoplasm of *β*‐catenin‐overexpressing RKO cells. J) WST‐1 assay illustrating that knocked down of *β*‐catenin in DLD1 and HCT15 cells abolished their sensitivity to NU2058 (72 h). Bars, SD; *p < 0.05; **p < 0.01; ***p < 0.001.

To explore the reason why multiple CRC cell lines displayed different responses to NU2058, the subcellular distribution of *β*‐catenin in the presence or absence of NU2058 was detected by Western blotting. Although NU2058 had no effect on the total *β*‐catenin level (**Figure** **2**A), the nuclear expression of *β*‐catenin was significantly decreased in DLD1 and HCT15 cells (Figure 2B), which was confirmed by immunofluorescence staining (Figure 2C). We did not observe a change in the total or subcellular expression of *β*‐catenin in NU2058‐treated RKO or SW620 cells (Figure 2A,B). Moreover, the mRNA expression of *cyclin D1* and *c‐Myc*, well‐known downstream genes of the Wnt/*β*‐catenin signaling pathway, was significantly reduced by NU2058 in DLD1 and HCT15 cells but not in RKO or SW620 cells (Figure 2D). The dual luciferase reporter assay showed that NU2058 significantly inhibited the transcriptional activity of the *cyclin D1* and *c‐Myc* promoters in DLD1 and HCT15 cells. When the transcription factor‐binding sites in the *cyclin D1* and *c‐Myc* promoters were mutated, the inhibitory effect of NU2058 on the transcriptional activity of *cyclin D1* and *c‐Myc* was markedly abolished (Figure 2E). The decreased cyclin D1 and c‐Myc expression in NU2058‐treated cells and tumor xenografts also proved that NU2058 suppressed CRC tumor growth by inhibiting the Wnt/*β*‐catenin pathway (Figure 2F,G). To further study the essential role of *β*‐catenin in the anticancer properties of NU2058, we forced the expression of *β*‐catenin in RKO cells and then performed cell viability assay in the presence of NU2058. Note that RKO cells overexpressing *β*‐catenin were more sensitive to NU2058 than were the control cells (Figure 2H,I). More importantly, the knockdown of *β*‐catenin obviously abolished the anticancer effect of NU2058 in DLD1and HCT15 cells (Figure 2J). These data suggest that NU2058 targets *β*‐catenin signaling to exert anticancer bioactivity. Furthermore, cell viability assay was performed to compare the anticancer effects of NU2058 with other *β*‐catenin inhibitors. The results showed that NU2058 exerted a better inhibitory effect on CRC cell proliferation, compared with ICRT3 and LF3 (Figure S2, Supporting Information).

### 2.3 RanBP3 Is a Direct Target of NU2058 and Suppresses CRC Tumorigenesis

**Figure 3:**
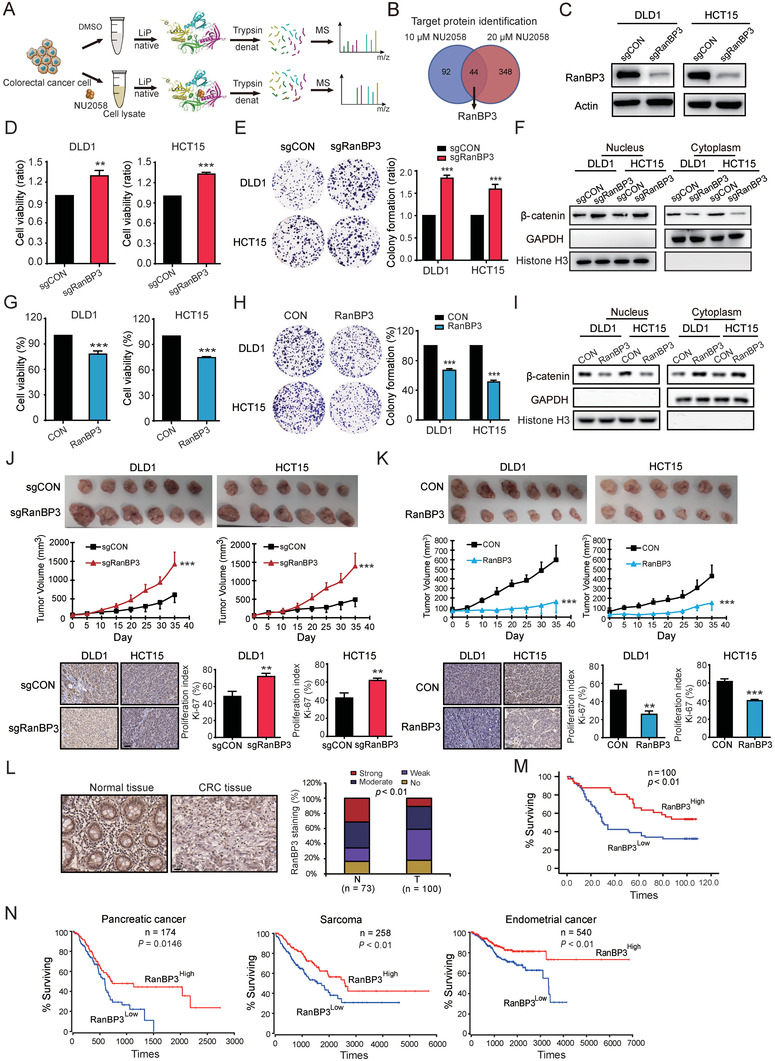
RanBP3 is a tumor suppressor and favorable prognostic biomarker in CRC. A) LiP‐SMap and mass spectrometry were used to identify the target proteins of NU2058. B) RanBP3 was proposed to be a direct target of NU2058. C) Successful knockout of RanBP3 in DLD1 and HCT15 cells. D,E) WST‐1 and colony formation assays were used to detect the effect of RanBP3 knockout on cell proliferation. F) Deletion of RanBP3 promoted the nuclear accumulation of *β*‐catenin in CRC cells. G,H) The effect of RanBP3 overexpression on cell proliferation was detected by WST‐1 and colony formation assays. I) Overexpression of RanBP3 reduced the nuclear accumulation of *β*‐catenin in CRC cells. J) Representative images and tumor curves showing that RanBP3 knockout promoted tumor growth (upper panel). Immunohistochemical analysis of Ki‐67 in tumor xenografts (lower panel). K) Tumor curve analysis and immunohistochemical staining of Ki‐67 showed that RanBP3 overexpression suppressed tumor growth. L) Representative images and expression pattern of RanBP3 in CRC tumor and adjacent normal tissues. M) Kaplan–Meier analysis showing the survival of 100 CRC patients based on tumor RanBP3 expression. N) The expression of RanBP3 predicted the survival of patients with pancreatic cancer, sarcoma, and endometrial cancer. Bars, SD; *p < 0.05; **p < 0.01; ***p < 0.001.

To decipher the molecular mechanisms by which NU2058 regulates the Wnt/*β*‐catenin pathway, LiP‐SMap coupled with MS was performed to identify the binding proteins of NU2058 (**Figure** **3**A,B). Among the 44 candidate proteins (Table S2, Supporting Information), RanBP3, which has been reported to enhance the nuclear export of active *β*‐catenin, drew our attention.^[^ ^12^ ^]^ To investigate the biological function of RanBP3 in CRC, CRISPR/Cas9 technology was used to generate RanBP3‐deficient CRC cells (Figure 3C). Knockout of RanBP3 not only markedly enhanced the abilities of CRC cells to proliferate and form colonies (Figure 3D,E) but also increased nuclear accumulation of *β*‐catenin and the expression of c‐Myc and cyclin D1 (Figure 3F and Figure S3A, Supporting Information), whereas overexpression of RanBP3 had the opposite effect (Figure 3G,I and Figure S3B, Supporting Information). Furthermore, the tumor xenografts established by RanBP3‐deficient DLD1‐sgRanBP3 and HCT15‐sgRanBP3 cells were significantly larger than those established by the control cells, with decreases in size of 62.8% and 66.2%, respectively. Ki‐67 immunostaining also showed that knockout of RanBP3 promoted the proliferation index of CRC tumor xenografts (Figure 3J). In contrast, overexpression of RanBP3 was found to suppress the proliferation of CRC cells and growth of CRC tumors in mice (Figure 3K).

To assess the clinical significance of RanBP3 in CRC, RanBP3 expression was detected in a tissue microarray containing 100 CRC tumor tissues and 73 corresponding normal tissues. As shown in Figure 3L, high RanBP3 expression was detected in 42.0% (42/100) of tumor tissues and 64.8% (47/73) of normal tissues. Moreover, there was a significant association between RanBP3 and N stage (**Table** **1**). Furthermore, Kaplan–Meier survival analysis revealed that the median survival time of patients with high RanBP3 expression (98 months) was significantly longer than that of patients with low RanBP3 expression (30 months) (Figure 3M). Analysis of data from OncoLnc (http://www.oncolnc.org/)^[^ ^13^ ^]^ confirmed that tumor RanBP3 expression could predict the survival of cancer patients (Figure 3N). In short, these data collectively suggest that RanBP3 is a tumor suppressor and favorable prognostic biomarker in CRC.

**Table 1:** Correlation between RanBP3 expression levels and clinicopathological parameters in 100 cases of colon cancer

Variable	*n*	Low RanBP3	High RanBP3	*p* value
Age [years]				
≤55	12	9	3	0.229
>55	88	50	38
Gender				
Female	44	25	19	0.694
Male	56	34	22
T‐Stage				
1/2	5	2	3	0.375
3/4	95	57	38
N‐Stage				
N0	62	31	31	0.019
N1	38	28	10
M‐Stage				
M0	98	57	41	0.134
M1	2	2	0
Pathologic stage				
Stages I and II	58	32	26	0.360
Stages III and IV	42	27	15

### 2.4 RanBP3 Is Essential for the Anticancer Bioactivity of NU2058 in CRC Cells

**Figure 4:**
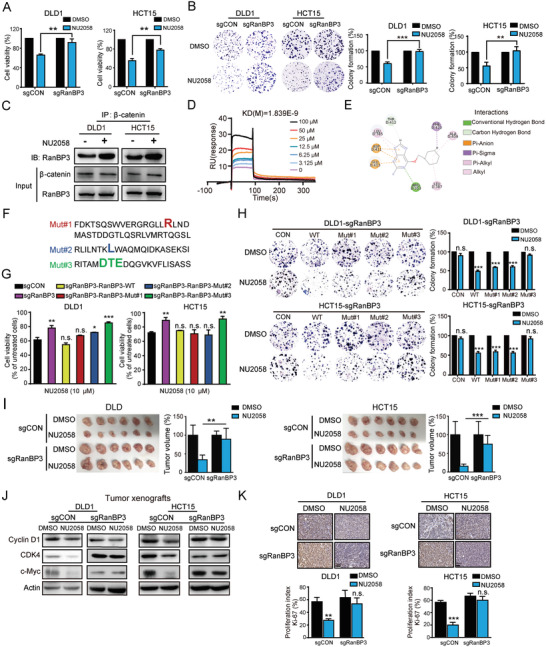
RanBP3 is a direct target of NU2058 and essential for the anticancer effect of NU2058 in CRC. A,B) Cell viability assay (A) and colony formation assay (B) were performed to determine the effect of NU2058 (0 and 10 µm) on the proliferation of RanBP3‐deficient cells. C) Co‐IP showing that the interaction between RanBP3 and *β*‐catenin was increased in the presence of NU2058. D) SPR assay showing the binding of NU2058 to the RanBP3 protein. E) The binding sites between NU2058 and RanBP3 were predicted by molecular docking. F) Schematic diagram of RanBP3 protein mutations. G,H) RanBP3‐WT, RanBP3‐Mut#1, RanBP3‐Mut#2, or RanBP3‐Mut#3 were expressed in RanBP3‐deficient cells, and the cells were then subjected to WST‐1 and colony formation assays. I) Tumor volumes and images showing that the anticancer bioactivity of NU2058 was abolished in tumor xenografts established from RanBP3‐deficient cells compared with those established from control cells (*n* = 6). J) Western blotting analysis of cyclin D1, CDK4, c‐Myc, and actin expression in the tumor xenografts. K) The Ki‐67 proliferation index was detected in NU2058‐treated tumor xenografts by immunohistochemistry (*n* = 3). Bars, SD; *p < 0.05; **p < 0.01; ***p < 0.001.

We next verified the role of RanBP3 in the anticancer effect of NU2058. WST‐1 and colony formation assays indicated that the anticancer effect of NU2058 was significantly abolished in RanBP3‐deficient DLD1 and HCT15 cells (**Figure** **4**A,B). It has been reported that RanBP3 is closely related to the nuclear export of *β*‐catenin. Immunoprecipitation (Co‐IP) was performed to detect the interaction of RanBP3 and *β*‐catenin in the presence of NU2058, and the results showed that NU2058 increased the RanBP3‐*β*‐catenin interaction in CRC cells (Figure 4C). In addition, the RanBP3 protein was purified, and surface plasmon resonance (SPR) assay was carried out to confirm the binding of NU2058 to RanBP3 (Figure 4D). The specific binding sites of NU2058 in RanBP3 were explored by molecular docking assay, and three RanBP3 mutant‐expressing plasmids were constructed accordingly (Figure 4E,F). RanBP3‐deficient DLD1 and HCT15 cells were further reinforced with wild‐type or three different mutant RanBP3 and then subjected to WST‐1 and colony formation assays in the presence or absence of NU2058. As shown in Figure 4G,H, NU2058 significantly inhibited the proliferation of RanBP3‐KO cells when wild‐type RanBP3, RanBP3 mutant #1, or RanBP3 mutant #2 was expressed but not when RanBP3 mutant #3 was expressed. The results suggested the essential role of asp‐406, thr‐407, and glu‐408 of RanBP3 in the anticancer properties of NU2058. Moreover, the importance of RanBP3 in the anticancer effect of NU2058 was evaluated in vivo. As shown in Figure 4I, NU2058 did not affect the tumorigenicity of RanBP3‐deficient cells in mice, although the growth of tumors derived from the control cells was obviously repressed. Similarly, the inhibitory effects of NU2058 on the *β*‐catenin pathway and Ki‐67 proliferation index in CRC tumor xenografts were markedly attenuated by RanBP3 knockout (Figure 4J,K).

### 2.5 NU2058 Selectively Induces Senescence of CRC Cells with Nuclear *β*‐Catenin Activation

**Figure 5:**
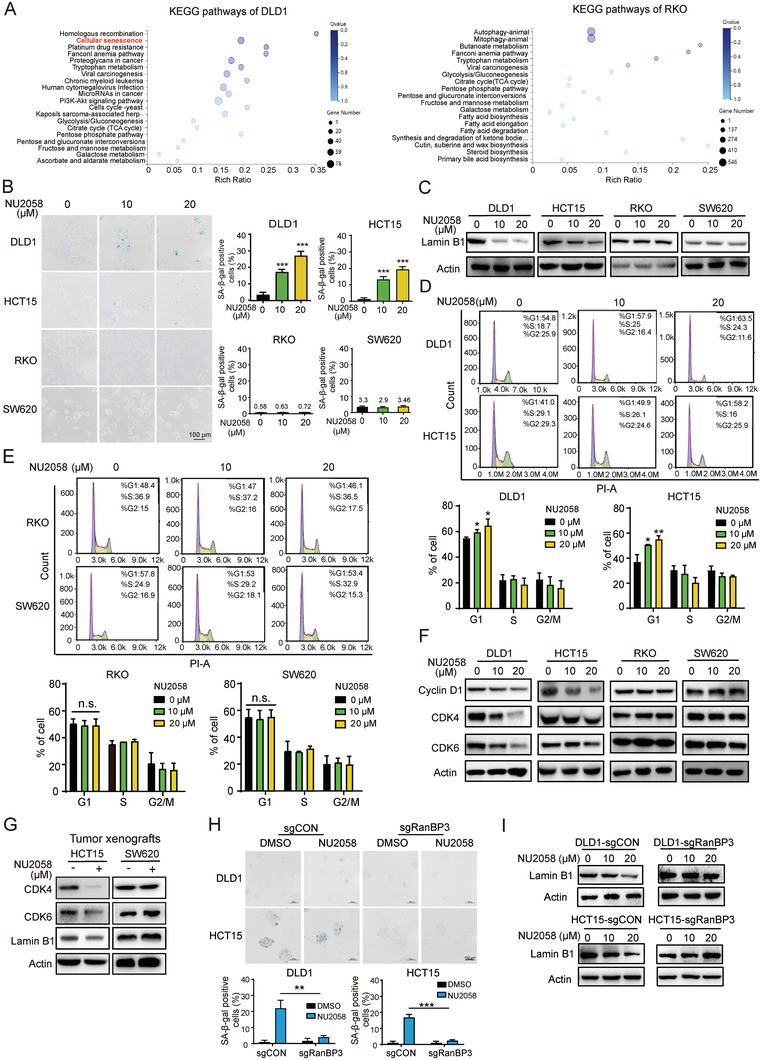
NU2058 induces cell senescence by directly targeting RanBP3‐*β*‐catenin signaling in CRC cells. A) RNA sequencing and KEGG pathway analysis suggested the involvement of cell senescence in the action mechanisms of NU2058 in CRC cells with nuclear *β*‐catenin activation. B) *β*‐Galactosidase staining and quantification showing that NU2058 (0, 10, and 20 µm, 72 h) induced senescence of DLD1 and HCT15 cells but not of RKO and SW620 cells. C) Expression of lamin B1 in NU2058‐treated CRC cells. D,E) The cell cycle distribution of DLD1, HCT15, RKO, and SW620 cells was analyzed by flow cytometry after exposure to increasing concentrations of NU2058 for 72 h. F) Western blotting showing the expression of cyclin D1, CDK4, CDK6, and actin in CRC cells treated with NU2058. G) Expression of CDK4, CDK6, lamin B1, and actin in tumor xenografts established from HCT15 and SW620 cells. H) *β*‐Galactosidase staining was performed to determine the effect of NU2058 on the senescence of RanBP3‐KO cells. I) RanBP3‐deficient cells were exposed to NU2058, and Western blotting was used to detect lamin B1 expression. Bars, SD; *p < 0.05; **p < 0.01; ***p < 0.001.

The above findings showing the anticancer effect of NU2058 led us to investigate whether NU2058 could induce apoptosis of CRC cells. Unexpectedly, we did not observe any apoptosis in NU2058‐treated cells (Figure S4A,B, Supporting Information). Therefore, RNA‐seq was performed to compare the gene profiles of CRC cells upon NU2058 treatment. As indicated by KEGG analysis, the differentially expressed genes in NU2058‐treated DLD1 cells, but not NU2058‐treated RKO cells, were significantly enriched in the cell senescence pathway, suggesting that cell senescence mediates the specific effect of NU2058 on CRC cells with nuclear *β*‐catenin activation (**Figure** **5**A). This idea was confirmed by positive *β*‐galactosidase staining in DLD1 and HCT15 cells exposed to NU2058, whereas no change was observed in RKO and SW620 cells (Figure 5B). The results from Western blotting also demonstrated that NU2058 decreased the expression of lamin B1, a key protein of cell senescence, in DLD1 and HCT15 cells but not in RKO and SW620 cells (Figure 5C). Cellular senescence is a process of permanent cell cycle arrest that suspends proliferating cells in the G1 phase of the cell cycle.^[^ ^14^ ^]^ Our results showed that NU2058 induced G1 arrest in DLD1 and HCT15 cells, but the cell cycle distribution did not change in RKO and SW620 cells (Figure 5D‐E). The expression levels of G1 phase‐related regulatory proteins were determined in the presence of NU2058, and we found dose‐dependent decreases in the expression of cyclin D1, CDK4, and CDK6 (Figure 5F). Similar results were observed in NU2058‐treated tumor xenografts (Figure 5G). Next, we examined whether RanBP3 participates in the effect of NU2058 on cell senescence. By assessing *β*‐galactosidase staining and lamin B1 expression, we found that NU2058‐induced cell senescence was significantly abolished in RanBP3‐KO cells, suggesting that NU2058 induces cell senescence by directly targeting RanBP3‐*β*‐catenin signaling (Figure 5H,I).

### 2.6 Preclinical Evaluation of NU2058 Either as a Single Agent or in Combination with Irinotecan and Oxaliplatin in CRC

**Figure 6:**
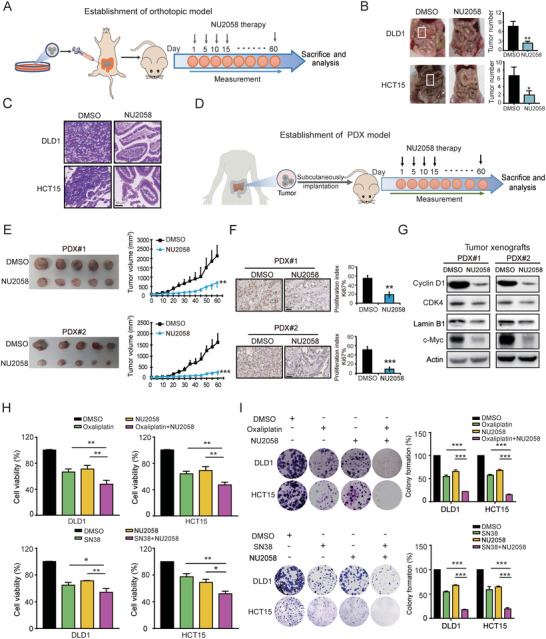
NU2058 suppresses CRC tumor growth in orthotopic xenograft models and PDX models and enhances sensitivity to SN38 and oxaliplatin. A) DLD1 and HCT15 cells were injected below the cecum to establish orthotopic xenograft models in nude mice, and the mice were given NU2058 (15 mg kg^−1^) or DMSO every 5 days (*n* = 5). B) Representative images and quantification of tumor nodules. C) Hematoxylin–eosin staining of mouse intestines. D) Schematic diagram showing the approach to establish patient‐derived xenografts (PDXs). E) Representative images and growth curves showing the growth of PDX tumors upon NU2058 treatment (*n* = 5). F) Immunohistochemical analysis of Ki‐67 in NU2058‐treated PDX tumors (*n* = 3). G) Western blotting was used to detect the expression of cyclin D1, CDK4, c‐Myc, and lamin B1 in PDX tumors. H,I) WST‐1 and colony formation assays suggested that NU2058 enhances the sensitivity of CRC cells to SN38 and oxaliplatin. Bars, SD; *p < 0.05; **p < 0.01; ***p < 0.001.

We further developed an orthotopic xenograft mouse model to evaluate the anticancer potential of NU2058. CRC cells were injected below the serosa of the cecum, and the mice were treated with NU2058 or DMSO (**Figure** **6**A). Our results showed that NU2058 remarkably inhibited the growth of orthotopic CRC cells, as evidenced by the numbers of tumor nodules (Figure 6B) and histological analysis of xenografts (Figure 6C). Furthermore, two PDX models were established by implanting tumor tissues from CRC patients into immunodeficient mice (Figure 6D). By monitoring the tumor size, we noted that NU2058 significantly delayed the growth of PDX tumors (Figure 6E), as evidenced by the reduced Ki‐67 proliferation index in the tumor xenografts (Figure 6F). Moreover, we assessed the downstream target genes of the Wnt/*β*‐catenin pathway in the PDX tumors, including cyclin D1, CDK4, c‐Myc, and lamin B, and the results supported that NU2058 treatment resulted in the inactivation of Wnt/*β*‐catenin signaling in vivo (Figure 6G). Drug resistance remains one of the major challenges in the clinical management of CRC.^[^ ^15^ ^]^ We further assessed the potential effects of NU2058 in CRC chemoresistance, and WST‐1 and colony formation assays were performed to examine the sensitivity of CRC cells to irinotecan (SN38) and oxaliplatin in the presence or absence of NU2058. As shown in Figure 6H,I, compared with treatment with NU2058, SN38 or oxaliplatin alone, the combined use of a low concentration of NU2058 and SN38 or oxaliplatin exerted a synergistic effect to suppress CRC tumorigenicity.

## 3 Discussion

LiP‐SMap is a reliable technique to identify target proteins of drugs.^[^ ^16^ ^]^ In this study, RanBP3 was identified as a direct target of NU2058. RanBP3 is a RanGTP‐binding protein that has been reported to enhance nuclear export of active *β*‐catenin, independent of its role as a CRM1‐associated nuclear export cofactor.^[^ ^17^ ^]^ However, the function and clinical significance of RanBP3 in CRC remain unknown. Here, we provide the first evidence suggesting that RanBP3 expression is downregulated in cancer tissues and can predict poor prognosis in CRC patients (Figure 3). More importantly, by gain‐ and loss‐of‐function studies, RanBP3 was found to significantly regulate CRC tumorigenesis. Furthermore, based on the results of molecular docking and SPR assays, we demonstrated that asp‐406, thr‐407, and glu‐408 of RanBP3 were essential for the binding of NU2058 to RanBP3 and the anticancer properties of NU2058.

**Figure 7:**
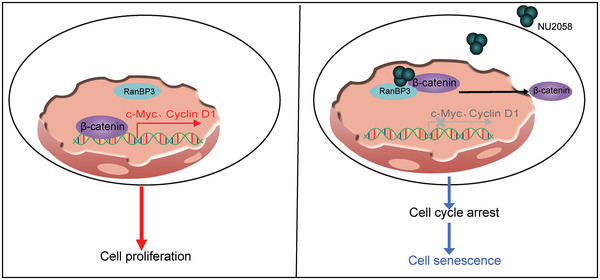
Schematic diagram summarizing how NU2058 suppresses tumorigenesis.

Senescence is commonly thought to function as a potential tumor‐suppressive mechanism; however, it may also promote tumor formation.^[^ ^18^ ^]^ Investigations of the complex regulatory mechanisms between Wnt/*β*‐catenin signaling and senescence in the development of cancer are ongoing.^[^ ^19^ ^]^ A recent study suggests that repression of Wnt/*β*‐catenin signaling is an early‐activated trigger of the senescence program.^[^ ^20^ ^]^ Another study suggested that there is a “tug of war” between cellular senescence and the tumor‐promoting activity of Wnt/*β*‐catenin signaling in early neoplasia.^[^ ^21^ ^]^ In the present study, by using RNA‐seq and a series of functional assays, we demonstrated that NU2058 disturbs the transcription of *cyclin D1* and *c‐Myc* to induce G1 cell cycle arrest and cell senescence, and this effect is dependent on RanBP3‐mediated nuclear translocation of *β*‐catenin (**Figure** **7**).

With the substantial advances in molecular oncology, extensive efforts have been made to investigate Wnt/*β*‐catenin signaling factors that can be inhibited; for example, the Wnt ligand/receptor interface, *β*‐catenin destruction complex, and TCF/*β*‐catenin transcription complex are considered potential targets in preclinical and clinical evaluations.^[^ ^22^ ^]^ Targeting the *β*‐catenin complex or *β*‐catenin nuclear translocation has emerged as an appealing approach.^[^ ^23^ ^]^ In the present study, our data illustrated that NU2058 could selectively suppress the tumorigenesis of CRC cells with nuclear *β*‐catenin activation in vivo and in vitro by enhancing the interaction of RanBP3 and *β*‐catenin to disturb the nuclear translocation of *β*‐catenin. The efficacy of NU2058 in CRC was also evaluated in a preclinical setting by using PDX and orthotopic models. Although we performed a series of in vitro and in vivo functional assays and various rescue experiments to demonstrate that *β*‐catenin is required for the anticancer property of NU2058, we could not exclude the possibility that other factors may be involved in the action mechanisms of NU2058, which warrants further investigation.

Oxaliplatin/SN38‐based therapy is the standard first‐line treatment for advanced or metastatic CRC; however, resistance to chemotherapy remains one of the major challenges in the clinical management of CRC.^[^ ^24^ ^]^ In this study, we also explored the effects of the combined use of NU2058 and oxaliplatin/SN38 in CRC and noted that NU2058 increased the sensitivity of CRC cells to oxaliplatin/SN38. Taken together, our results suggest the potential of NU2058 in the treatment of CRC, particularly for patients with activation of nuclear *β*‐catenin.

In conclusion, our preclinical study supports the potential therapeutic application of NU2058 for the treatment of CRC patients with nuclear *β*‐catenin activation as a strategy to combat Wnt‐driven CRC.

## 4 Experimental Section

### Cell Culture and Drug

The human CRC cell lines DLD1, HCT15, RKO, and SW620 were purchased from ATCC (Rockville, MD, USA), and the normal colonic epithelial cell line NCM460 was obtained from INCELL Corporation (San Antonio, TX, USA). The cells were cultured with DMEM (Thermo Fisher Scientific, San Jose, CA, USA) containing 10% FBS (ExCell Bio, Shanghai, China) in a 5% CO_2_ cell incubator at 37 °C. NU2058 was purchased from Selleck Chemicals (Huston, TX, USA) and dissolved in DMSO.

### Plasmids, Transfection, Infection, and Site‐Directed Mutagenesis

The coding sequences of human *β*‐catenin and wild‐type or mutant RanBP3 were amplified and cloned into the pcDNA3.1 vector. The wild‐type and mutant promoter regions of c‐Myc and cyclin D1 were cloned into the pGL3 vector (Promega, Madison, WI, USA). Mutations in the c‐Myc and cyclin D1 promoters and RanBP3 were created by the Fast Mutagenesis System (TransGen Biotech, Beijing, China). Small interference RNAs (siRNAs) against *β*‐catenin (Transheep) were transfected into DLD1 and HCT15 cells by Lipofectamine 3000 (Thermo Fisher Scientific). The sequences of the primers used for mutation are listed in Table S3, Supporting Information, and the target sequences of siRNAs for *β*‐catenin are listed in Table S4, Supporting Information.

### Cell Viability Assay

Cells were seeded into a 96‐well plate with 3000 cells per well, and different concentrations of drugs were added after 24 h. Cell viability was measured by a WST‐1 Cell Proliferation and Cytotoxicity Assay Kit (Beyotime, Shanghai, China).^[^ ^25^ ^]^ The absorbance was measured at 450 nm with a microplate spectrophotometer (BioTek Instruments, Winooski, VT, USA).

### Colony Formation Assay

Colony‐formation assay was performed as described previously.^[^ ^26^ ^]^ Approximately 3000 cells were seeded into a 6‐well plate and treated with NU2058 or other drugs for 14 days. The cells were washed with PBS, fixed with methanol for 10 min and then stained with 1% crystal violet, and the number of colonies was counted for analysis.^[^ ^27^ ^]^

### Cell Cycle Analysis

Cells were fixed with 70% ethanol and stained with propidium iodide/RNase (Beyotime) staining solution at 37 °C for 30 min. The cell cycle was analyzed by flow cytometry (BD Biosciences, San Diego, CA, USA).

### Cell Apoptosis Analysis

Cell apoptosis assay was performed following the instructions of the Annexin V‐PE kit (KeyGen, Nanjing, China). Cells were resuspended in 300 µL PBS and incubated with 3 µL propidium iodide and 3 µL Annexin V‐FITC at room temperature for 20 min. Apoptotic cells were detected by flow cytometry (BD Biosciences).

### Western Blotting Analysis

The cell lysates were prepared as previously described,^[^ ^28^ ^]^ and the protein concentration was determined with a BCA kit (Thermo Fisher Scientific). Protein samples were separated by sodium dodecyl sulfate (SDS)‐PAGE and transferred to PVDF membranes (Millipore, Bedford, MA, USA). After blocking with 5% fat‐free milk for 1 h, the PVDF membrane was incubated with primary antibody for 1–2 h at room temperature and then washed with 1× tris‐buffered saline with Tween (TBST) for 30 min. Next, the membrane was incubated with the corresponding horseradish peroxidase‐conjugated secondary antibodies for 1 h. After washing with TBST, signals were detected using ECL substrate (Bio–Rad, Hercules, CA, USA). The antibodies used included actin and RanBP3 from Santa Cruz Biotechnology (Santa Cruz, CA, USA), CDK4 and p‐Rb from Cell Signaling Technology (Beverly, MA, USA), cyclin D1, CDK6, GAPDH, histone H3, lamin B1, *β*‐catenin, and c‐Myc from Proteintech (Rosemont, IL, USA).

### Senescent Cell Staining

Cell senescence was detected by a senescence *β*‐galactosidase staining kit (Beyotime). The adherent cells were incubated with fixative buffer for 15 min and stained with working staining solution containing *β*‐galactosidase substrate at 37 °C for 14 h. The percentage of positively stained cells was calculated.

### Subcellular Fractionation

Cells were incubated with extraction buffer containing protease inhibitor and Triton‐100 for 30 min, and nuclear isolation buffer containing sucrose was added to the mixture. Then, the cell nuclear pellet was collected by centrifugation. Acetone was added to the supernatant to precipitate cytoplasmic protein, and finally, the cytoplasmic protein was dissolved in SDS lysis buffer. The expression of nuclear proteins and cytoplasmic proteins was verified by Western blotting. Lamin B1/histone H3 and GAPDH were used as nuclear and cytosolic loading controls, respectively.

### Immunofluorescence

As previously described,^[^ ^16b^ ^]^ cells were treated with paraformaldehyde for 10 min and blocked with 5% BSA for 1.5 h after treatment with Triton X‐100. Next, the cells were incubated with primary antibody overnight at 4 °C, washed with TBST, and then incubated with the corresponding fluorescent secondary antibody for 1.5 h at room temperature. After counterstaining with DAPI (Beyotime), images of the stained cells were obtained by laser scanning confocal microscopy.

### Dual Luciferase Reporter Assay

The dual luciferase reporter assay was performed by using the Dual‐Luciferase Reporter Assay System (Promega, Madison, WI, USA).^[^ ^29^ ^]^ Cells were cotransfected with a luciferase plasmid expressing the *β*‐catenin promoter region and a pRL‐TK Renilla luciferase‐expressing plasmid and then treated with NU2058 for 72 h, and luciferase activity was measured according to the manufacturer's instructions.

### Reverse Transcription and Quantitative Real‐Time Polymerase Chain Reaction

The process used for quantitative real‐time polymerase chain reaction (qRT–PCR) has been described elsewhere.^[^ ^30^ ^]^ Total RNA was extracted by TRIzol reagent (Life Technologies), and cDNA was synthesized with a Primescript First‐Strand cDNA Synthesis kit (Takara, Dalian, China) according to the manufacturer's instructions. The mRNA expression levels of GAPDH, c‐Myc, and cyclin D1 were detected by qPCR. The primer list is shown in Table S5, Supporting Information.

### LiP‐SMap

The cell lysate was mixed with different concentrations of NU2058, subjected to limited proteolysis with proteinase K (PK), and then completely digested with trypsin under degenerative conditions to generate peptides for mass spectrometry assessment.^[^ ^31^ ^]^ The peptide was freeze‐dried in vacuum and resuspended in anhydrous acetonitrile solution, followed by desalination with a MonoTip C18 Pipette Tip (GL Sciences, Tokyo, Japan). Peptide samples were analyzed and identified by an Orbitrap Fusion Lumos mass spectrometer (Thermo Fisher Scientific). Finally, the raw data were analyzed by Proteome Discoverer (Thermo Fisher Scientific) and Spectronaut software (Omicsolution Co., Ltd., Shanghai, China) to identify targets of NU2058.

### Tissue Microarray and Immunohistochemistry

Immunohistochemistry was carried out as previously described.^[^ ^29^ ^]^ The expression of RanBP3 was analyzed in a tissue microarray containing 100 CRC tissues and 73 normal tissues (Shanghai Outdo Biotech, Shanghai, China). In brief, the tissue microarray was incubated with primary antibody against RanBP3 (1:100 dilution, Santa Cruz) overnight at 4 °C, followed by incubation with the corresponding biotinylated secondary antibody. The signals were divided into four groups: score 0 (negative), score 1 (weakly positive), score 2 (moderately positive), and score 3 (strongly positive). Scores of 0 and 1 were classified as low expression, while scores of 2 and 3 were considered high expression.

### CRISPR/Cas9‐Mediated Gene Knockout

Gene knockout was performed by using CRISPR/Cas9 technology. The annealed sgRNA oligos were cloned into the lentiCRISPRv2 vector (Addgene plasmid #52961), a gift from Feng Zhang (Massachusetts Institute of Technology Cambridge, MA, USA),^[^ ^32^ ^]^ to generate the RanBP3‐knockout plasmid. 293T cells were cotransfected with the RanBP3‐knockout plasmid and packaging plasmids, and DLD1 and HCT15 cells were then infected with the virus‐containing supernatant. RanBP3‐deficient cells were selected by puromycin, and RanBP3 deficiency was confirmed by Western blotting and genomic DNA sequencing. The sequence of the sgRNA targeting RanBP3 was TCCAGTCCTGAAGGCGGAGA.

### Purification of the RanBP3 Protein

The full‐length RanBP3 coding sequence was amplified and cloned into the prokaryotic expression vector pGEX‐6P1 with a glutathione S‐transferase (GST) tag and then transformed into *Escherichia coli* BL21. When the O.D. value of the bacterial culture reached 0.6–0.8, 0.5 µm isopropyl *β*‐d‐thiogalactopyranoside (IPTG) was added, and the sample was cultured at 37 °C for 4 h. After sonication, the supernatant was collected by centrifugation at 12 000 × *g* for 30 min. GST‐tagged RanBP3 fusion protein was isolated with a GST‐tagged protein purification kit (Beyotime). Finally, the GST tag was removed by PreScission Protease (Beyotime).

### SPR

The interaction between RanBP3 and NU2058 was detected using a Biacore X100 system (GE Healthcare Life Sciences, Marlborough, MA, USA) as described previously.^[^ ^33^ ^]^ RanBP3 was immobilized onto an active CM7 chip (GE Healthcare Life Sciences). Different concentrations of NU2058 were flowed through the CM7 chip for 10 min with a flow rate of 30 µL min^−1^ to bind proteins, and then the Kd value was analyzed.

### Co‐IP

Immunoprecipitation was performed as described previously.^[^ ^34^ ^]^ CRC cells were cotransfected with *β*‐catenin‐ and RanBP3‐expressing plasmids in the presence of NU2058. The cell lysate was incubated with IgG (Cell Signaling Technology) and protein A/G Sepharose beads (Invitrogen) at 4 °C for 1 h. After centrifugation, the supernatant was mixed with the appropriate primary antibody overnight at 4 °C, and then protein A/G Sepharose beads were added and incubated for 4 h. After washing with PBS three times, the beads were mixed with 5× SDS‐PAGE loading buffer for Western blotting analysis.

### RNA‐Seq

RNA‐seq was performed at Beijing Genomics Institute Tech (Shenzhen, Guangzhou, China). Gene profiles were analyzed by the DESeq R software package (1.10.1), and genes with a fold change >2 were deemed differentially expressed genes.^[^ ^2^ ^]^

### Tumor Xenograft Experiment

The tumor xenograft experiment was performed as described previously.^[^ ^29^, ^30^ ^]^ BALB/c nude mice aged 6–8 weeks were fed under standard conditions according to institutional guidelines. A total of 2 × 0^6^ cells were resuspended in a mixture of PBS and Matrigel at equal volumes, and the cell suspension was subcutaneously injected into the flanks of the mice. The tumor size and body weight of the mice were measured every 3 days, and the tumor volume was calculated as *V* = (length × width^2^)/2. For the drug treatment experiments, a total of 5 × 10^5^ cells were subcutaneously injected into the flanks of the mice. When the tumor diameter reached ≈5 mm, the mice were randomly divided into a treatment group and a control group. The treatment group was given 15 mg kg^−1^ NU2058 by intragastric administration every day, while the control group received vehicle control. Finally, the tumor, liver, lung, and kidney were collected for Western blotting and histological analyses.

### Orthotopic Tumor Model

BALB/c nude mice aged 6–8 weeks were anesthetized and placed in the supine position. A midline incision was made to exteriorize the cecum. 2 × 10^6^ DLD cells or HCT15 cells were resuspended in a mixture of PBS and Matrigel. The cell mixture was injected into the base of the cecum. Then, the cecum was returned to the peritoneal cavity, and the incision was sutured. The mice were divided into two groups and treated with 15 mg kg^−1^ NU2058 or vehicle control twice a week. All mice were sacrificed 2 months after the surgery, and then the intestines were collected to measure the number of tumor nodules.

### PDX Model

Fresh tumor tissues were isolated from the two CRC patients, cut into 0.5–1 mm^3^ sections in sterile conditions, and then subcutaneously inoculated into NOD‐Prkdc^scid^‐Il2rg^em1IDMO^ mice (Beijing IDMO Co., Ltd., Beijing, China).^[^ ^35^ ^]^ When the diameter of the tumor reached 5 mm, the mice were divided into two groups and treated with NU2058 or vehicle control twice a week, and the tumor size was measured every 5 days.

All animal experiments were conducted in accordance with the rules and regulations of the Ethics Committee for Animal Experiments of Guangzhou Medical University (G2022‐085).

### Statistical Analysis

All in vitro experiments were performed as three independent experiments, and the data were expressed as the means ± SD. The significance of differences was calculated using Student's‐*t* test in GraphPad Prism software (San Diego, CA, USA), and *p* values <0.05 were considered significantly different.

## Conflict of Interest

The authors declare no conflict of interest.

## Author Contributions

C.‐C.Z., L.L., and Y.‐P.L. contributed equally to this work. C.‐C.Z., L.L., and Y.‐P.L.: acquisition of data, analysis and interpretation of data, statistical analysis, drafting of the manuscript; Y.‐M.Y., Y.H., G.‐G.Z., and S.‐J.L.: acquisition of data; T.L.: critical revision of the manuscript for important intellectual content; B.L. and W.W.X.: funding acquisition, study concept and design, study supervision.

## Supporting information

Supporting Information

## Data Availability

The data that support the findings of this study are available in the supplementary material of this article.
