# Supplementary material for: Blockade of Nuclear β‐Catenin Signaling via Direct Targeting of RanBP3 with NU2058 Induces Cell Senescence to Suppress Colorectal Tumorigenesis
Source: Adv Sci (Weinh). 2022 Oct 21;9(34):2202528. doi: 10.1002/advs.202202528 (PMC9731691; doi:10.1002/advs.202202528)
Supplement: Supplementary file 1 — Supporting Information [file ADVS-9-2202528-s001.pdf]

ADVANCED SCIENCE
Open Access

Supporting Information

for *Adv. Sci.*, DOI 10.1002/advs.202202528

Blockade of Nuclear β-Catenin Signaling via Direct Targeting of RanBP3 with NU2058 Induces Cell Senescence to Suppress Colorectal Tumorigenesis

Can-Can Zheng, Long Liao, Ya-Ping Liu, Yan-Ming Yang, Yan He, Guo-Geng Zhang, Shu-Jun Li, Ting Liu, Wen Wen Xu and Bin Li**

Supplementary Figure legends

Supplementary Figure 1. (A) The body weight of nude mice exposed to NU2058 was monitored. (B) Representative images of the lung, liver, kidney tissues stained by H&E staining. (C) Serum alanine transaminase activity (ALT), serum aspartate transaminase activity (AST), hemoglobin (HGB), red blood cells (RBC), platelets (PLTs), lymphocyte levels were detected in NU2058-treated and control groups (n = 3). (D) The cell proliferation of KYSE410 and Huh7 cells was suppressed by NU2058. Bars, SD; *, $P < 0.05$; **, $P < 0.01$; ***, $P < 0.001$.

Supplementary Figure 2. Comparison of the anticancer effects of NU2058 with other β-Catenin inhibitors in DLD1 and HCT15 cells. Bars, SD; *, $P < 0.05$; **, $P < 0.01$; ***, $P < 0.001$.

Supplementary Figure 3. (A-B) Western blotting showing the expression of c-Myc and cyclin D1 in the RanBP3-knockout cells (A) and RanBP3-overexpressing cells (B).

Supplementary Figure 4. (A) Analysis of cell apoptosis upon NU2058 treatment by flow cytometry. (B) Western blotting showing the expression of apoptosis markers in NU2058-treated CRC cells. Bars, SD; *, $P < 0.05$; **, $P < 0.01$; ***, $P < 0.001$.

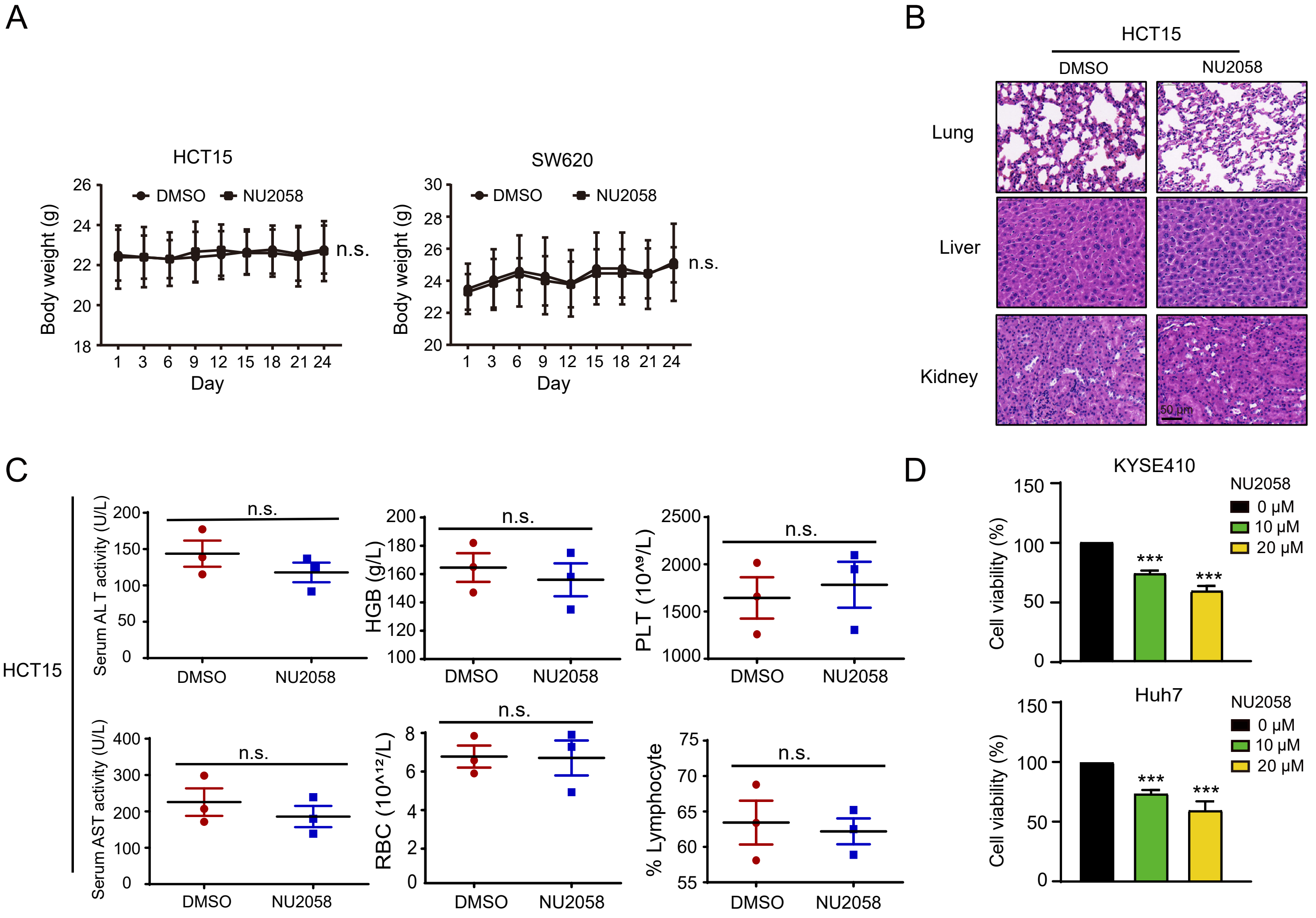

Supplementary Figure 1

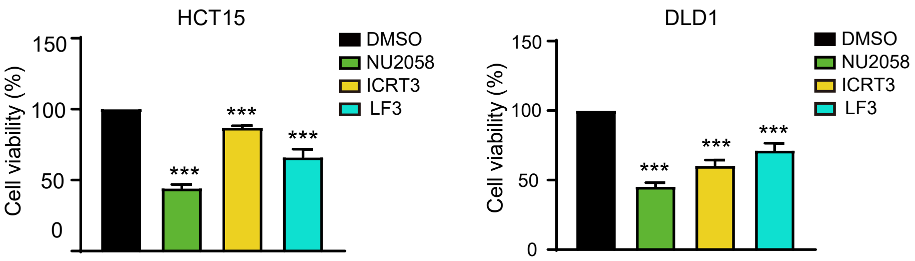

Supplementary Figure 2

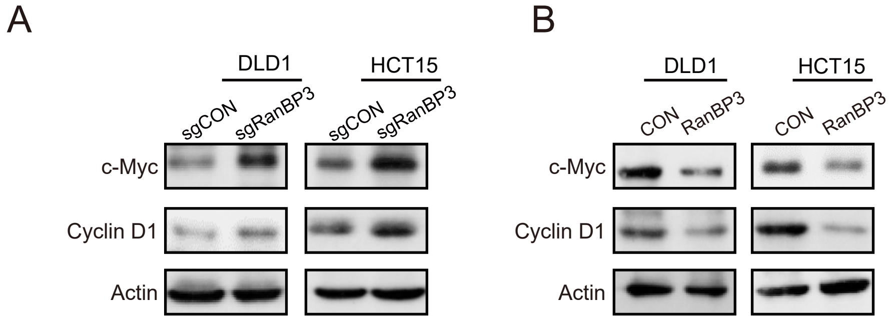

Supplementary Figure 3

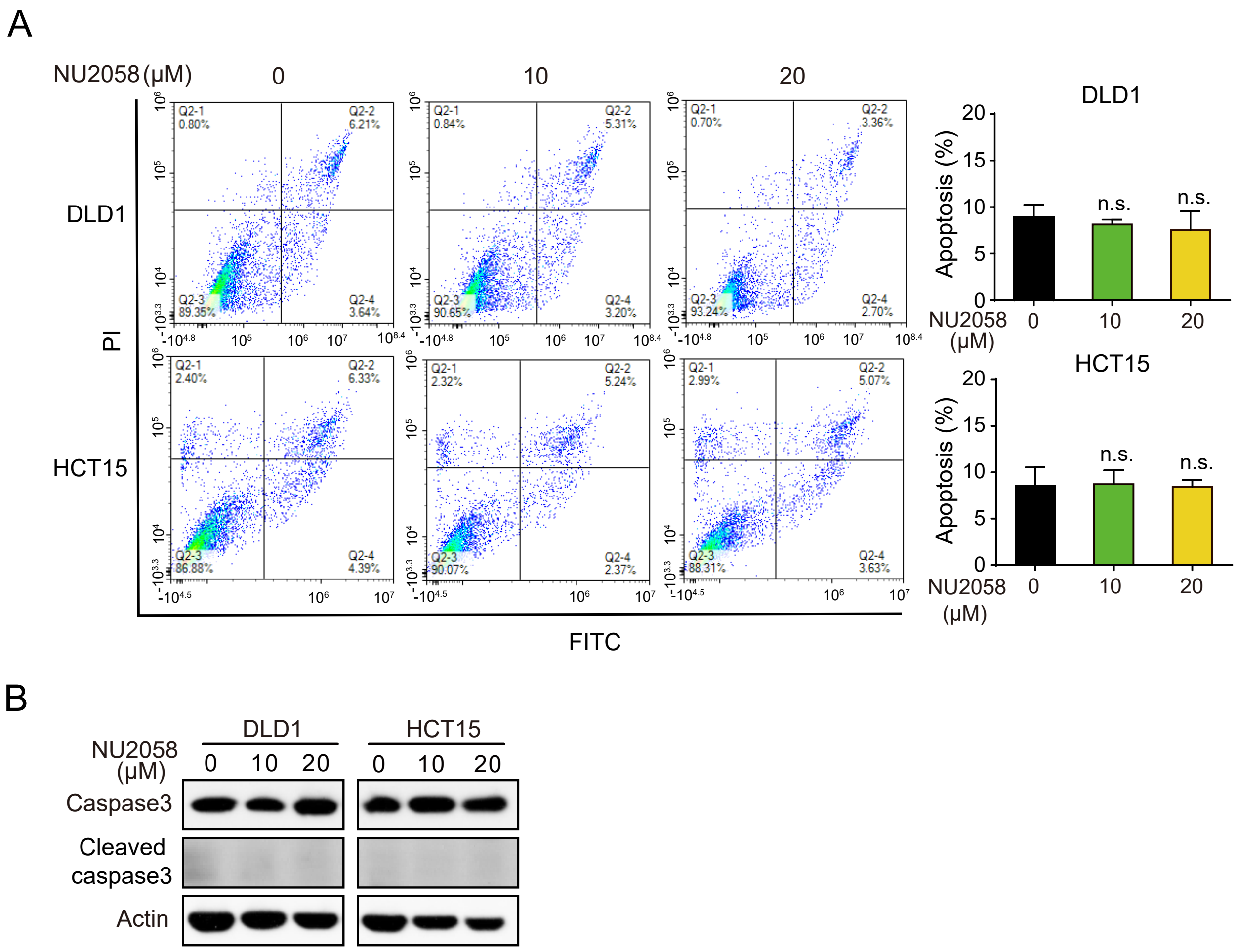

Supplementary Figure 4

Supplementary Table 1. The effect of 280 drugs on CRC cell proliferation.

	**Compound**	**Cell viability of DLD1 (%)**	**Cell viability of RKO (%)**	**Cell viability of NCM460 (%)**
1	Defactinib (VS-6063, PF-04554878)	100.5	67.9	103.8
2	CL-387785 (EKI-785)	31.9	36.0	87.8
3	1NM-PP1	89.6	94.2	115.0
4	2-Chloropyrazine	97.8	100.9	123.8
5	NSC 42834	92.0	91.4	120.5
6	Methyl hesperidin	96.1	97.3	118.4
7	Lithium citrate tribasic tetrahydrate	100.2	101.1	102.2
8	2,3-Butanedione 2-Monoxime	96.6	98.0	102.5
9	2-Methoxy-1,4-naphthoquinone	20.4	26.6	37.1
10	Ruxolitinib (INCB-18424) phosphate	101.8	100.7	96.7
11	Oxfendazole	97.8	45.4	94.6
12	Cyanoacetohydrazide	100.7	105.6	130.0
13	Adenosine 5'-monophosphate monohydrate	103.5	106.2	130.2
14	Phenformin hydrochloride	103.6	106.7	123.5
15	Nilotinib (AMN-107)	102.8	61.3	119.9
16	Y-27632 dihydrochloride	102.6	106.7	127.0
17	PIK75	10.0	26.1	19.7
18	Tofacitinib (CP-690550) Citrate	101.3	103.7	103.3
19	TG100-115	100.0	104.6	105.4
20	Amlexanox	101.8	102.1	113.1
21	HTH-01-015	100.3	68.8	116.4
22	SKF-86002	103.7	108.9	123.7
23	RGB-286638 free base	21.5	38.1	37.9
24	LY2409881 trihydrochloride	88.5	42.9	113.6
25	PQ401	105.1	108.1	121.5
26	XL147 analogue	79.6	86.3	104.3
27	CEP-32496	55.3	28.3	88.0
28	INK 128 (MLN0128)	32.6	27.9	48.5
29	AZD2932	89.5	110.6	103.7
30	BX795	79.4	63.6	85.0
31	BX912	57.4	36.6	105.6
32	SB202190 (FHPI)	104.2	101.6	123.3
33	Temsirolimus (CCI-779, NSC 683864)	86.5	66.7	119.3
34	PF 431396	38.3	34.9	34.0
35	PIK-294	102.8	102.4	121.5

36	SGX-523	104.3	95.6	124.4
37	HS-173	29.7	42.1	33.6
38	SBI-0206965	49.1	41.0	82.7
39	CNX-774	74.8	92.8	70.4
40	BS-181	99.4	92.4	99.8
41	K 02288	88.2	53.9	126.9
42	PLX647	76.1	51.2	85.9
43	VX-702	104.4	79.9	99.0
44	IRAK-1/4 Inhibitor	104.1	99.4	123.8
45	SBE 13 hydrochloride	104.3	102.5	122.5
46	IM-12	71.1	59.6	121.4
47	GSK2578215A	97.9	97.7	85.9
48	Losmapimod (GW856553X)	99.5	89.7	104.5
49	LRRK2-IN-1	94.2	45.0	94.5
50	TCS PIM-1 1	100.7	100.5	100.3
51	CX-4945 (Silmitasertib)	56.8	71.9	125.5
52	BLU9931	83.1	66.0	91.8
53	ANA-12	93.3	98.4	123.0
54	Decernotinib (VX-509)	103.8	108.0	124.7
55	Dacomitinib (PF299804, PF299)	27.1	43.1	55.6
56	TAS-301	99.9	94.6	127.0
57	Palomid 529 (P529)	91.7	58.6	84.1
58	Pyridone 6	78.0	92.6	99.5
59	Fasudil (HA-1077) hydrochloride	100.0	106.2	105.8
60	SMI-4a	102.0	104.2	101.4
61	AS601245	88.0	74.9	112.2
62	CGK 733	38.6	31.1	26.8
63	TBB (NSC 231634)	98.7	101.9	123.7
64	ZCL 278	99.8	98.6	122.5
65	Daphnetin	98.6	90.6	125.1
66	7,8-Dihydroxyflavone	97.2	98.3	127.2
67	Ginsenoside Rg2	98.0	97.9	110.0
68	Lapatinib (GW-572016) Ditosylate	71.9	59.5	104.8
69	Palbociclib (PD-0332991) hydrochloride	81.2	60.0	97.1
70	Palbociclib (PD0332991) Isethionate	81.1	57.6	96.5
71	TSU-68 (SU6668, Orantinib)	93.7	92.6	125.4
72	LY2835219 mesylate	45.8	45.5	32.7
73	Forsythin	91.4	98.5	115.4
74	Balicatib	94.2	95.1	112.6
75	BIX02188	91.9	85.3	118.6
76	CX-6258	63.3	6.4	5.0

77	(R)-Ruxolitinib (INCB018424)	96.5	97.0	99.0
78	ZM 336372	95.0	81.5	103.5
79	Afatinib (BIBW2992) Dimaleate	12.1	29.9	23.8
80	6-BIO	6.5	1.1	18.9
81	XL184 (Cabozantinib malate)	14.6	22.6	74.7
82	Apitolisib (GDC-0980, RG7422)	31.0	22.9	46.4
83	Afuresertib	50.6	34.5	28.1
84	Acalisib (GS-9820)	87.8	63.1	97.3
85	NVP-BVU972	91.7	71.0	94.0
86	BMS-265246	79.7	62.2	93.9
87	PF-04217903	96.7	87.6	98.5
88	ML167	89.7	82.0	95.1
89	JNK-IN-8	77.7	42.8	91.3
90	WH-4-023	80.9	83.5	99.2
91	WHI-P258	87.8	98.1	98.5
92	TH588	57.7	42.6	52.9
93	GNF-5	102.9	105.0	97.9
94	ISRIB	109.9	95.5	101.3
95	WHI-P180	87.3	66.3	100.0
96	Oclacitinib	105.5	96.2	98.5
97	RKI-1313	111.2	91.3	93.7
98	GDC-0032	72.6	37.6	89.6
99	ZM 323881 hydrochloride	98.5	89.4	100.6
100	LY2090314	107.3	79.9	67.6
101	PD153035 hydrochloride	4.9	25.4	76.0
102	ZM 306416	100.7	106.6	103.0
103	KU-60019	106.5	57.6	108.0
104	PHT-427	109.2	108.5	102.8
105	R406 free base	99.7	86.4	99.3
106	SKLB1002	108.7	105.5	102.4
107	ID-8	105.5	110.3	100.9
108	PF-562271	32.9	42.9	37.1
109	Torkinib (PP242)	48.3	31.2	64.9
110	EHop-016	23.1	43.2	36.2
111	Binimetinib (MEK162, ARRY-162, ARRY-438162)	88.1	28.5	98.6
112	ZM 39923 hydrochloride	109.3	102.7	102.2
113	D 4476	105.8	109.6	103.1
114	PIK-90	87.3	63.0	99.9
115	KI8751	68.5	69.9	93.0
116	GZD824 Dimesylate	0.9	24.6	28.0
117	AZD5438	34.3	35.9	23.2
118	MK8745	70.0	72.5	83.6

119	PP121	28.3	26.7	24.8
120	SL-327	103.4	53.1	100.7
121	MLN0905	76.7	47.3	38.5
122	SB590885	110.4	55.8	103.8
123	CID755673	106.8	98.0	102.1
124	PF-06447475	105.7	115.2	103.9
125	WS3	22.8	42.8	24.2
126	WS6	35.8	47.5	26.7
127	CGI1746	86.9	108.6	94.8
128	CC-292 (AVL-292)	32.9	48.4	72.2
129	CEP-33779	98.8	52.1	96.2
130	BIX02189	82.4	58.4	92.8
131	Baricitinib (LY3009104, INCB028050)	103.5	110.9	99.5
132	AZ5104	23.4	52.0	31.1
133	PRT062607 (P505-15, BIIB057) hydrochloride	64.8	33.1	72.9
134	AT13148	103.0	72.8	94.3
135	Tideglusib	103.3	92.4	99.8
136	PD-173955	76.4	43.4	88.8
137	GSK2334470	67.5	37.6	93.6
138	GSK2126458(Omipalisib)	10.5	44.6	37.1
139	Thiazovivin	103.5	92.1	95.7
140	R788 (Fostamatinib) Disodium	88.1	71.5	93.6
141	KN-93 Phosphate	98.7	120.1	97.1
142	CHIR-98014	109.1	117.0	95.7
143	CCT137690	17.3	41.0	36.4
144	GSK2606414	53.3	62.4	85.9
145	SNS-314 Mesylate	46.5	38.9	86.0
146	CH5132799	59.2	24.3	80.8
147	SGI-1776 free base	8.3	42.8	32.2
148	GLPG0634 analogue	86.3	76.4	93.9
149	Gandotinib (LY2784544)	42.5	45.6	71.4
150	UNC2881	84.7	75.4	86.2
151	G-749	0.5	29.9	22.0
152	PF670462	90.9	74.2	93.0
153	Poziotinib (HM781-36B)	66.8	60.3	45.4
154	CHIR98014	88.0	44.8	54.5
155	KRCA-0008	63.5	53.1	58.1
156	GZD 824	1.8	12.8	23.8
157	GSK429286A	101.8	97.2	95.1
158	GSK2656157	84.3	85.8	89.9
159	RO 3280	71.2	40.1	47.0

160	OSI 930	101.1	89.0	94.2
161	GW 2580	88.4	98.0	93.4
162	Flavopiridol (Alvocidib) hydrochloride	22.2	31.3	19.3
163	PIK-93	60.0	27.9	79.6
164	IC-87114	93.6	94.2	95.8
165	AG18	91.6	85.0	99.9
166	APY0201	16.5	29.6	33.7
167	kb NB 142-70	96.5	92.0	100.7
168	AZD7545	90.7	67.8	87.9
169	ML347	44.4	48.2	48.3
170	LDN-214117	90.5	56.9	102.3
171	CZC24832	93.0	95.6	95.3
172	MK-2206 dihydrochloride	88.9	48.2	87.8
173	L-779,450	98.0	43.4	93.4
174	SRPIN340	99.1	88.0	91.7
175	AZD2858	105.0	92.7	89.9
176	AZ20	36.9	38.5	31.0
177	PND-1186	71.9	27.7	81.9
178	ASP 3026	76.5	49.9	86.5
179	INCB28060 (Capmatinib)	100.6	89.5	100.5
180	AZD3463	27.8	19.9	28.2
181	BYL-719 (Alpelisib)	63.7	28.5	81.9
182	Filgotinib (GLPG0634)	100.2	96.5	87.1
183	LDN212854	1.9	36.5	23.3
184	MHY1485	98.9	98.8	90.6
185	NVP 231	30.0	36.6	49.9
186	PH797804	103.6	97.2	99.6
187	TAE226 (NVP-TAE226)	22.3	25.3	69.9
188	RN486	103.2	89.6	100.8
189	BML-275	101.3	96.0	102.2
190	AT 7519 hydrochloride salt	37.7	33.9	27.2
191	SKLB 610	87.5	102.3	72.9
192	RKI-1447	105.1	71.5	85.6
193	TG003	100.2	90.2	81.4
194	U0126-EtOH	99.0	72.5	92.5
195	AMG-337	95.8	88.9	97.4
196	URMC-099	10.7	46.4	35.3
197	CH5183284 (Debio-1347)	71.3	47.5	75.4
198	FRAX597	19.5	68.1	87.4
199	Ro 31-8220 Mesylate	32.4	37.3	26.1
200	CVT-313	71.5	79.9	94.4
201	YM201636	53.9	42.0	28.0

202	GW 441756	98.8	76.4	65.9
203	R112	40.3	35.4	86.3
204	GNF-7	41.9	28.1	76.0
205	VX-11e	75.8	28.1	90.8
206	SZL P1-41	103.7	94.2	101.5
207	Carbazochrome	102.6	99.8	101.3
208	Gypenoside XVII	104.7	96.1	101.0
209	Momordin Ic	103.2	97.7	102.0
210	NU7441 (KU-57788)	91.6	50.8	99.7
211	MRT67307	80.7	49.5	78.0
212	Solcitinib	97.8	94.4	91.0
213	Ensartinib	65.3	35.7	72.1
214	GNE-0877	96.8	85.0	94.0
215	Ddr1-In-1	87.5	43.5	49.2
216	Sodium houttuyfonate	101.3	87.6	96.7
217	Mubritinib (TAK 165)	98.7	100.0	89.9
218	Kaempferitrin	102.4	88.8	99.5
219	AZ 960	70.5	48.7	71.9
220	CZC-25146	70.6	63.5	85.1
221	JNK-IN-7	26.6	45.4	30.4
222	NQDI-1	98.7	93.2	87.4
223	IQ-1S	94.2	79.7	79.3
224	ETC-1002	94.3	85.6	91.5
225	BAW2881	91.8	66.7	86.7
226	SC66	23.6	39.0	30.8
227	MLN1117	79.5	50.3	92.4
228	AZD-9291 mesylate	25.1	33.9	29.8
229	AZD3759	83.0	81.4	98.0
230	K858	31.9	42.4	49.8
231	Dimethylenastron	24.5	37.1	31.9
232	Mollugin	56.5	44.2	86.6
233	Hydroxysafflor yellow A	85.6	87.3	93.0
234	Ruboxistaurin hydrochloride	69.0	51.1	33.3
235	GNE 477	24.5	15.4	47.5
236	AM-2394	75.7	86.2	95.4
237	HUHS015	37.8	45.7	55.6
238	NU2058	58.2	86.4	90.7
239	CC-223	41.3	28.7	65.6
240	XMD8-87	58.0	89.2	101.7
241	XMD16-5	21.2	45.4	93.5
242	Selonsertib	87.4	81.0	97.1
243	SCH900776 S-isomer	73.6	43.5	71.6
244	MCC950	90.9	85.7	100.2

245	GDC-0084	58.7	41.1	90.1
246	GNE-3511	81.6	71.7	94.1
247	SUN 11602	92.9	86.1	102.0
248	BI-78D3	64.3	63.5	101.7
249	magnolin	86.8	96.8	102.6
250	Olivetol	93.9	88.9	101.0
251	ML281	101.7	63.3	101.8
252	Rolapitant hydrochloride	92.8	86.8	103.3
253	KD025 (SLx-2119)	59.8	45.2	44.5
254	Uprosertib (GSK2141795)	71.9	40.3	78.1
255	NVP-BSK805 2HCl	62.2	17.1	27.7
256	MCB-613	19.9	38.3	34.8
257	BI-847325	10.0	41.3	38.2
258	WZ4003	92.1	98.0	101.2
259	CC-115	27.3	26.2	34.1
260	ARQ-092	100.6	91.3	105.6
261	GSK583	88.4	75.7	106.7
262	Olmutinib (HM61713, BI 1482694)	76.9	90.4	98.3
263	PIK-III	81.8	70.6	100.4
264	PF-543	85.7	110.4	108.6
265	AZD8186	78.7	58.9	99.7
266	FIIN-2	79.5	89.0	97.0
267	DEL-22379	38.0	35.8	49.6
268	CCG215022	97.0	99.1	105.4
269	Desmethylanethol trithione	100.3	98.4	103.2
270	eFT508	96.0	104.7	103.2
271	SB239063	96.0	92.9	107.3
272	APS-2-79	82.5	103.5	101.5
273	CHIR-124	11.7	39.6	31.0
274	Pamapimod (R-1503, Ro4402257)	90.5	86.6	100.4
275	GSK180736A	99.2	106.2	106.4
276	Ulixertinib (BVD-523, VRT752271)	59.3	25.3	102.9
277	JI-101	89.6	94.5	93.5
278	GDC-0879	95.2	38.6	100.8
279	BMS-582949 HCl	95.1	91.5	97.4
280	NIH-12848	95.9	101.8	100.0

Supplementary Table 2. The binding proteins of NU2058 was identified by Limited proteolysis-small molecule mapping (Lip-SMap).

	Entry	Protein Descriptions	Protein Names
1	Q8TBF2	Prostamide/prostaglandin F synthase	PXL2B
2	P56937	3-keto-steroid reductase	DHB7
3	A0FGR8	Extended synaptotagmin-2	ESYT2
4	Q9UI10	Translation initiation factor eIF-2B subunit delta	EI2BD
5	P62854	40S ribosomal protein S26	RS26
6	P19447	General transcription and DNA repair factor IIH helicase subunit XPB	ERCC3
7	Q8IW19	Aprataxin and PNK-like factor	APLF
8	Q96DG6	Carboxymethylenebutenolidase homolog	CMBL
9	B2RTY4	Unconventional myosin-Ixa	MYO9A
10	Q969U7	Proteasome assembly chaperone 2	PSMG2
11	P46779	60S ribosomal protein L28	RL28
12	Q9H6Z4	Ran-binding protein 3	RANB3
13	Q7L014	Probable ATP-dependent RNA helicase DDX46	DDX46
14	Q96EU7	C1GALT1-specific chaperone 1	C1GLC
15	O15511	Actin-related protein 2/3 complex subunit 5	ARPC5
16	Q13163	Dual specificity mitogen-activated protein kinase kinase 5	MP2K5
17	Q9Y478	5'-AMP-activated protein kinase subunit beta-1	AAKB1
18	Q14680	Maternal embryonic leucine zipper kinase	MELK
19	Q9Y5X2	Sorting nexin-8	SNX8
20	O75122	CLIP-associating protein 2	CLAP2
21	Q8NBA8	DTW domain-containing protein 2	DTWD2
22	Q15233	Non-POU domain-containing octamer-binding protein	NONO
23	Q53G59	Kelch-like protein 12	KLH12
24	P63220	40S ribosomal protein S21	RS21
25	Q9Y6I9	Testis-expressed protein 264	TX264
26	P52655	Transcription initiation factor IIA subunit 1	TF2AA
27	A8MXV4	Nucleoside diphosphate-linked moiety X motif 19	NUD19
28	Q9Y6X5	Bis(5'-adenosyl)-triphosphatase ENPP4	ENPP4
29	P08779	Keratin, type I cytoskeletal 16	K1C16
30	Q53EP0	Fibronectin type III domain-containing protein 3B	FND3B
31	O00212	Rho-related GTP-binding protein RhoD	RHOD
32	Q96P47	Arf-GAP with GTPase, ANK repeat and PH domain-containing protein	AGAP3
33	Q9UBL3	Set1/Ash2 histone methyltransferase complex subunit ASH2	ASH2L
34	Q8WUJ0	Serine/threonine/tyrosine-interacting protein	STYX
35	P27338	Amine oxidase [flavin-containing] B	AOFB
36	Q53GL7	Poly [ADP-ribose] polymerase 10	PAR10
37	Q9NQC3	Reticulon-4	RTN4
38	Q6UX07	Dehydrogenase/reductase SDR family member 13	DHR13
39	P00338	L-lactate dehydrogenase A chain	LDHA
40	Q9UKM9	RNA-binding protein Raly	RALY
41	Q9NVX2	Notchless protein homolog 1	NLE1
42	O75368	SH3 domain-binding glutamic acid-rich-like protein	SH3L1
43	Q9BVL4	Selenoprotein O	SELO
44	O95415	Brain protein I3	BRI3

Supplementary Table 3. The list of primers used for mutation design.

**Primer name**	**Sequence**
pcDNA3.1^{+}RanBP3^{R354G}-F	5'-AGAGGCCGGGGGCTGCTCGGACTCAATGA-3'
pcDNA3.1^{+}RanBP3^{R354G}-R	5'-CGAGCAGCCCCCGGCCTCTCTCCACCCA-3'
pcDNA3.1^{+}RanBP3^{L386G}-F	5'-ATCCTCAACACCAAGGGGTGGGCCCAGAT-3'
pcDNA3.1^{+}RanBP3^{L386G}-R	5'-CCCTTGGTGTTGAGGATCAGTCGCAGGCT-3'
pcDNA3.1^{+}RanBP3$^{D406G, T407G, E408G}$-F	5'-CACAGCCATGGGCGGCGGGGACCAGGGCGT-3'
pcDNA3.1^{+}RanBP3$^{D406G, T407G, E408G}$-R	5'-CCGCCGCCCATGGCTGTGATGCGAATGCTCT-3'
c-Myc-promoter site1$_{mut}$-F	5′-AGTGTATCAGAATCGATGCATTTTTTG-3'
c-Myc-promoter site1$_{mut}$-R	5′-ATACACTAAGAGGAGAGGAGTATTAC-3'
c-Myc-promoter site2$_{mut}$-F	5′-CCCTGGCTTCTCCCAAACCCGGCAG-3'
c-Myc-promoter site2$_{mut}$-R	5′-GCCAGGGGTGCTAGACGGGAGAATATG-3'
cyclinD1-promoter site1$_{mut}$-F	5′-ACTCTATCTTTGCTTAACAACAGTAAC-3'
cyclinD1-promoter site1$_{mut}$-R	5′-GATAGAGTCCCGGCAGAGAATGGGAG-3'
cyclinD1-promoter site2$_{mut}$-F	5′-TTACCAGTCCTGGAGCCTCCAGAGG-3'
cyclinD1-promoter site2$_{mut}$-R	5′-CTGGTAAACTTCAACAAAACTCCCCT-3'

Supplementary Table 4. The target sequences of siRNA against β-catenin.

**siRNA**	**Target sequence**
si-β-catenin-F	5'-GUUAUCAGAGGACUAAAUATT-3'
si-β-catenin-R	5'-UAUUUAGUCCUCUGAUAACTT-3'

Supplementary Table 5. The primers of GAPDH, c-Myc and cyclin D1.

**Primer name**	**Sequence**
GAPDH-F	5′-CGGAGTCAACGGATTTGGTCGTAT-3′
GAPDH-R	5′-AGCCTTCTCCATGGTGGTGAAGAC-3′
c-Myc-F	5′-CCTGGTGCTCCATGAGGAGAC-3′
c-Myc-R	5′-CAGACTCTGACCTTTTGCCAGG-3′
Cyclin D1-F	5′-TCTACACCGACAACTCCATCCG-3′
Cyclin D1-R	5′-TCTGGCATTTTGGAGAGGAAGTG-3′
